# Imaging the therapeutic effects of cerebral endothelial cell derived exosome treatment of a preclinical model of Alzheimer’s disease

**DOI:** 10.3389/fnagi.2026.1788077

**Published:** 2026-08-18

**Authors:** Lian Li, Guangliang Ding, Li Zhang, Hao Luo, Edward Boyd, Qingjiang Li, Jasleen Kaur, Manisha Bohara, Michael Chopp, Zheng Gang Zhang, Quan Jiang

**Affiliations:** 1 Department of Neurology, Henry Ford Health, Detroit, MI, United States; 2 Department of Physics, Oakland University, Rochester, MI, United States

**Keywords:** Alzheimer’s disease, CEC-Exo, CSF motion, glymphatic transport, MRI, structural integrity

## Abstract

**Background:**

Alzheimer’s disease (AD) is characterized by the excessive accumulation of select proteins in the brain, associated with altered vascular function, glymphatic transport, cerebrospinal fluid (CSF) dynamics, and cerebral microstructure. Exosomes derived from cerebral endothelial cells (CEC-Exo) have been demonstrated to reduce vascular dysfunction, which may beneficially impact perivascular glymphatic clearance, and potentially, protein aggregates and the corresponding microstructural disruption.

**Methods:**

We investigated the effect of CEC-Exo therapy on AD by assessing glymphatic transport, CSF motion, and microstructural integrity using magnetic resonance imaging (MRI), and measuring cognitive performance. TgF344-AD rats, at 12-months of age, were randomly treated with CEC-Exo (1×10^11^ particles/intravenous injection) or saline twice-weekly for four consecutive weeks. MRI measurements, including dynamic 3D T1-weighted imaging with contrast agent and diffusion MRI (dMRI) with multiple *b*-values, were conducted for all rats at 13-months. Glymphatic function was evaluated with tracer-induced time signal curve (TSC), while microstructural state and CSF motion were assessed with parameters derived from dMRI acquired at high (dMRI_high-b_) and low (dMRI_low-b_) *b*-values, respectively. Cognitive performance was assessed by the Morris water maze (MWM) test.

**Results:**

TSCs demonstrated significantly higher peak values in the olfactory bulb and whole brain in the CEC-Exo-treated group than in the saline-treated group. With the CEC-Exo treatment, a significantly increased MD_low-b_, a measure of pseudorandom CSF motion derived from dMRI_low-b_, was observed in the representative CSF-filled space. As revealed by dMRI_high-b_-derived parameters, CEC-Exo treatment resulted in significantly elevated axonal water fraction, fractional anisotropy, and Kurtosis in white matter, as well as significantly reduced mean diffusivity, axial diffusivity, and radial diffusivity in both white and gray matter in the AD brain. In the MWM test, CEC-Exo-treated rats spent a significantly greater percentage of time in the correct quadrant than did saline-treated rats.

**Conclusion:**

CEC-Exo treatment preserved glymphatic transport, CSF motion, and microstructural integrity in the AD brain, accompanied by a reduced cognitive decline. With a global therapeutic impact, the CEC-Exo therapy provides a novel strategy on the treatment of AD. Our data support the value of dMRI_low-b_ as a clinically feasible imaging tool for assessing alterations in CSF dynamics that mediate perivascular glymphatic clearance.

## 1 Introduction

As a progressive neurodegenerative disorder, Alzheimer’s disease (AD) is characterized by the excessive accumulation of proteins [e.g., amyloid-*β* (Aβ)] in the brain, attributed largely to impaired cerebral waste clearance that removes proteins and metabolites from the brain (Bloom, 2014; Yin et al., 2021; Zhang et al., 2021). Despite therapeutic efforts targeting Aβ deposition and its efflux (Sperling et al., 2011; Da Mesquita et al., 2018; Da Mesquita et al., 2021; Jäkel et al., 2022), to date, effective strategies are lacking (Sousa et al., 2023) for counteracting the brain damage [e.g., vascular (Solis et al., 2020; Soto-Rojas et al., 2021; Fisher et al., 2022; Alkhalifa et al., 2023) and structural (Snow et al., 2017; Dong et al., 2020; Collij et al., 2021; Zhao et al., 2025) disruption] caused by extracellular protein aggregates and compromised cerebral waste clearance.

By continuous movement through the complex interconnected space in the central nervous system (CNS), cerebrospinal fluid (CSF) plays an important role in draining metabolites generated by neural activities (Simon and Iliff, 2016; Matsumae et al., 2019; Nazeri et al., 2025). Accumulating evidence indicates that multiple efflux routes contribute to CSF drainage into extracranial lymphatic vessels (Kaur et al., 2021; Li et al., 2022b; Licastro et al., 2024). Among these CSF-mediated clearance pathways is the glymphatic system, a glia-dependent brain-wide network of perivascular channels, through which CSF penetrates the brain parenchyma via the periarterial space, mixes with interstitial fluid (ISF), and then exits the brain via the perivenous space (Iliff et al., 2012). This perivascular CSF flow in the brain parenchyma is impacted by CSF motion in the subarachnoid space (SAS), which accounts for an important component of the glymphatic transport (Harrison et al., 2018; Chen et al., 2024; Rane Levendovszky et al., 2024). Dysfunction of glymphatic transport and abnormalities in CSF dynamics have been implicated in various neurodegenerative disorders (Tazoe et al., 2023; Chen et al., 2024; Licastro et al., 2024; Pierobon Mays et al., 2024; Ran et al., 2024), including AD (Lahiri and Ray, 2013; Schubert et al., 2019; Nikolenko et al., 2020; Yankova et al., 2021).

In addition to the intricate and diverse nature of neurological disorders, effective drug delivery to the CNS is often restricted due to the presence of the blood–brain barrier (BBB). Exosomes are small lipid extracellular vesicles and active biological containers that mediate intercellular communication by transferring proteins and genetic information and instructions between cells (Marcus and Leonard, 2013; Schneider and Simons, 2013; Taylor and Gercel-Taylor, 2013). These nanosized extracellular vesicles, released by all cell types, have emerged as novel therapeutic agents and drug carriers for the treatment of brain diseases owing to their ability to cross the BBB, their specific molecular contents, and their capacity to be engineered (Hill, 2019; Choi et al., 2024). Exosomes derived from different cells have exhibited therapeutic effects in mitigating the symptoms of neurodegenerative disorders by slowing down Aβ aggregation, decreasing Aβ pathologies, attenuating neuroinflammation, preserving neuronal integrity, ameliorating destructive structural changes, and alleviating neurodegeneration (Yuyama et al., 2014; Yuyama et al., 2015; Cui et al., 2019; You and Ikezu, 2019; Ge et al., 2020; Guo et al., 2020; Guy and Offen, 2020; Soares Martins et al., 2021; Choi et al., 2024; Halipi et al., 2024; Lin et al., 2024). There are multiple ongoing clinical trials (Phases 1 and 2) investigating exosome-based therapies for neurological diseases, including stroke (NCT03384433), diabetes (NCT02138331), dementia (NCT05326724) and AD (NCT04388982) (Lotfy et al., 2023; Sanadgol et al., 2025), while in-depth exploration of specific exosomes and treatment strategies is ongoing. Our previous studies demonstrate that treatment of stroke and diabetes with exosomes derived from healthy cerebral endothelial cells (CEC-Exo) substantially reduces cerebrovascular damage and cognitive deficits (Li et al., 2021; Ding et al., 2022; Zhang et al., 2022). Neurovascular dysfunction is among the common pathogenic mechanisms (Steen et al., 2005; De Felice and Ferreira, 2014) that diabetes (Yan et al., 2020; Feng and Gao, 2024) and AD (Soto-Rojas et al., 2021; Fisher et al., 2022) share. The therapeutic effect of CEC-Exo on cerebrovascular integrity could beneficially impact perivascular glymphatic transport, which is dependent on the condition of cerebral vessels (Verheggen et al., 2018; Yankova et al., 2021; Licastro et al., 2024). However, whether CEC-Exo therapy ameliorates glymphatic dysfunction in the AD brain, thereby lowering the accumulation of harmful substances and their associated cerebral microstructural degeneration (Dong et al., 2020; Bazydlo et al., 2022; Sun et al., 2025), remains unexplored.

Diffusion magnetic resonance imaging (dMRI) acquired at high *b*-values (dMRI_high-b_) reveals important information about cerebral microstructure (Li et al., 2019; Wei et al., 2021), while dMRI obtained at low *b*-values (dMRI_low-b_) identifies the pseudo-diffusion characteristics of CSF motion (Fujiwara et al., 2025; Nazeri et al., 2025). With sensitivity and specificity to cerebral microstructural integrity, Gaussian and non-Gaussian diffusion parameters derived from dMRI_high-b_ (such as fractional anisotropy (FA) and Kurtosis, respectively) have been widely used to detect the subtle changes in tissue microstructure following brain insult and corresponding therapy (Adamson et al., 2013; Li et al., 2019; Wen et al., 2019; Wei et al., 2021; Chu et al., 2022; Zhang et al., 2025). Prior work has shown that CSF effective motility, which includes both laminar and/or mixing flow, can be characterized by its pseudo-diffusivity, a measure of pseudorandom CSF motion magnitude derived from dMRI_low-b_ (Nazeri et al., 2025). With dMRI_low-b_, the features of CSF motion within the ventricular system (Chen et al., 2024; Ran et al., 2024), along the perivascular spaces (Harrison et al., 2018; Wen et al., 2022; Evans et al., 2024; Ran et al., 2024), and in the SAS (Chen et al., 2024; Rane Levendovszky et al., 2024) have been investigated in both human and animal models. In addition to the prominent CSF motion near large arteries (Chen et al., 2024) and at the ventral surface of the brainstem (Matsumae et al., 2019; Taoka et al., 2021), previous studies have demonstrated that altered CSF motion is associated with aging (Taoka et al., 2021; Wen et al., 2022; Ran et al., 2024; Rane Levendovszky et al., 2024), sleep deprivation (Rane Levendovszky et al., 2024), cardiovascular (Evans et al., 2024) and neurodegenerative (Tazoe et al., 2023; Chen et al., 2024; Pierobon Mays et al., 2024; Ran et al., 2024) conditions. Without administration of CSF tracer, dMRI_low-b_ thus offers a non-invasive, non-disruptive method to quantify variations in CSF motion pertaining to brain diseases. However, whether dMRI_low-b_-derived measure of CSF motion is sensitive to a therapeutic intervention (such as CEC-Exo therapy) that potentially improves glymphatic transport in the AD brain remains unknown. Moreover, while a substantial number of studies have demonstrated altered glymphatic transport (Jiang et al., 2017; Reeves et al., 2020; Ang et al., 2024; Licastro et al., 2024) or CSF motion (Lahiri and Ray, 2013; Schubert et al., 2019; Tazoe et al., 2023; Pierobon Mays et al., 2024) under pathological conditions, how they concomitantly respond to treatment has been insufficiently investigated.

The primary objective of this study is to image the effect of CEC-Exo therapy on the function and structure of the AD brain. We propose to test the hypothesis that the vascular benefits of CEC-Exo could beneficially impact glymphatic transport. In addition to dynamic contrast-enhanced MRI (DCE-MRI), which is typically used for evaluating glymphatic function, we employed dMRI with multiple *b*-values ranging from 0 to 1,800 s/mm^2^ to assess CSF motion and microstructural integrity in a rat model of AD. By focusing on both the brain parenchyma and SAS, this is, to our knowledge, the first *in vivo* imaging study to concurrently reveal the changes in glymphatic transport, CSF motion, and cerebral microstructure in the AD brain resulting from CEC-Exo treatment. Our data, with and without image tracer, demonstrated that CEC-Exo therapy augmented glymphatic transport, elevated CSF motion, and reduced microstructural damage in the AD brain, accompanied by slowed cognitive decline. The consistent therapeutic responses found in both CSF motion in the SAS and glymphatic transport in the brain parenchyma evidenced their expected relevance and highlighted the sensitivity of dMRI_low-b_ to alterations in CSF dynamics in the AD brain that underlie Aβ accumulation, microstructural disruption, and cognitive deficits.

## 2 Materials and methods

All experimental procedures were approved by the Institutional Animal Care and Use Committee of Henry Ford Health and carried out in accordance with the National Institutes of Health (NIH) Guide for the Care and Use of Laboratory Animals. To avoid bias in the study, investigators responsible for collecting and analyzing outcome data (e.g., MRI acquisition, image processing, data analysis, and behavioral testing) remained blinded to group status throughout the experimental procedures.

### 2.1 Animals, CEC-Exo and experimental procedures

Transgenic rats that express amyloid precursor protein (APP) and presenilin 1 (PS1) mutations (line TgF344-AD), and manifest age-dependent cerebral amyloidosis between 6 and 26 months in conjunction with cognitive impairment (Cohen et al., 2013) were used in the present study. TgF344-AD rats (12-months; Rat Resource and Research Center, Columbia, MO, USA), with and without CEC-Exo treatment, were subjected to the identical experimental procedures for imaging study and cognitive assessment.

Primary CECs were isolated from cerebral microvessels of healthy adult F344 WT rats (3–4-months, Charles River) according to published method (Teng et al., 2008). Using differential ultracentrifugation, the CEC-Exo were isolated from culture medium of passages 1 to 2 CECs following previously described protocol (Li et al., 2021). The particle numbers and size distribution of CEC-Exo were characterized by a nanoparticle tracking analyzer (NTA, NanoSight N300 System, Merkel Technologies, Israel). Ultra-structural morphology and protein markers of CEC-Exo were examined by means of transmission electron microscopy (TEM) following transmission electron microscopy and Western blot, respectively. NTA revealed that particles isolated from CECs had an average size of 70.5 ± 1.1 nm, which is within the typical size range of exosomes (Chopp and Zhang, 2015). TEM and Western blot analysis demonstrated that CEC-Exo had cup-shaped morphology and expressed Exo membrane marker proteins including Alix and CD63, but not the intracellular protein calnexin, respectively.

The rats were randomly assigned to either the AD-Control or AD-Treated group (*n* = 10/group), with equal numbers of males and females in each group. Using the same CEC-Exo treatment protocol that has shown significant therapeutic effects on cerebrovascular function in stroke and diabetes (Ding et al., 2022; Zhang et al., 2022), rats in the AD-Treated group received twice-weekly intravenous administration of CEC-Exo at concentration of 1 × 10^11^ particles per injection for a total of four consecutive weeks, starting at the age of 12-months. In the AD-Control group, rats were treated with the same volume of saline as in the AD-Treated group. Given the hypothesis that CEC-Exo would provide similar therapeutic effects on cerebrovascular function in AD as observed in stroke and diabetes, the dose and treatment regimen used in those established models were employed in the current study. All animals were monitored twice weekly for general health (e.g., body weight, posture, breathing, coat condition) and for any visible adverse reactions to the repeated intravenous treatments. No observable adverse events, including injection site reactions, lethargy, or respiratory distress, were noted in any rats. No early mortality occurred over the 4-week treatment period. Furthermore, no significant reduction in body weight was detected in rats treated with CEC-Exo or saline. Collectively, these data indicate that CEC-Exo treatment is well-tolerated in AD rats.

MRI scans were conducted for all AD rats in both groups at 13-months. For CSF tracer infusion during DCE-MRI, prior to MRI, a catheter was implanted into the cisterna magna of the brain (Ding et al., 2018) for each animal studied. Briefly, the rats were initially anesthetized by inhalation of 3% isoflurane and maintained at 1.0–1.5% isoflurane in a mixture of N_2_O (70%) and O_2_ (30%) via a nose mask throughout the surgical procedure. Rectal temperature was maintained at 37 ± 1 °C using a feedback-regulated water heating system. The head of the anesthetized rat was mounted in a stereotactic frame with care to permit spontaneous breathing. After the atlanto-occipital membrane was exposed using a midline dorsal neck incision, a polyethylene catheter (PE-10 tubing; Becton Dickinson, MD, US) filled with saline was inserted into the subarachnoid cisterna magna space via a small durotomy made with a 27-gage needle. The outside part of the catheter was fixed to the occipital bone with superglue, and the skin incision was closed around the catheter.

MRI was performed with a 7 T system (Bruker-Biospin, Billerica, MA, USA) (Ding et al., 2018). A birdcage type coil was used as the transmitter and a quadrature half-volume coil as the receiver. An initial bolus of dexmedetomidine [15 μg/kg (Benveniste et al., 2017)] was injected using a Harvard pump into a pre-implanted subcutaneous catheter. The animal was placed in a prone position and securely fixed on an MR-compatible holder equipped with an adjustable nose cone for anesthetic gas administration and stereotaxic ear bars to immobilize the head. For reproducible positioning of the animal in the magnet, a fast gradient-echo imaging sequence, which produces three orthogonal slice images, was used at the beginning of each MRI session. During image acquisition, anesthesia was maintained by a continuous infusion of dexmedetomidine at 15–20 μg/kg/h (Benveniste et al., 2017) and a gas mixture of N_2_O (70%) and O_2_ (30%) with 0.1–0.2% isoflurane (Piramal Inc., Bethlehem, PA, USA). Respiratory rate was monitored using an air pad sensor (Biopac Systems Inc., Goleta, CA, USA), and rectal temperature was maintained at 37 ± 1 °C using a feedback-controlled air heating blower (Rapid Electric, Brewster, NY, USA).

To detect changes in cerebral microstructure and CSF motion, diffusion-weighted images (DWIs) using a single-shot spin-echo echo planar imaging diffusion sequence with 20 diffusion-encoding directions and multiple *b-*values (0, 50, 80, 120, 150, 180, 900, and 1,800 s/mm^2^) were acquired. Other imaging parameters were TR/TE = 10,000/50 ms, *δ*/Δ = 10/18 ms, FOV = 32 × 32 mm^2^, matrix = 128 × 128, number of averages = 2. Fifteen axial slices were collected with a 0.2 mm gap and a slice thickness of 0.8 mm.

To monitor the dynamic influx and clear-out process, 3D T1-weighted imaging (T1WI) (echo time (TE) = 4 ms, repetition time (TR) = 18 ms, flip angle = 12°, field of view (FOV) = 32 × 32 × 16mm^3^, matrix = 256 × 192 × 96) with the contrast agent Gd-DTPA was acquired after diffusion measurement. The time series of T1WI scanning continued for 5 h, starting with three baseline scans followed by intra-cisterna magna delivery of Gd-DTPA (21 mM concentration) at a constant infusion rate of 1.6 μL/min over 50 min (Jiang et al., 2017; Ding et al., 2018) via the indwelling catheter connected to a 100 μL syringe (Hamilton Robotics, Reno, NV, US) mounted on an infusion pump (Harvard Apparatus, Holliston, MA, US).

### 2.2 MRI data processing

The detailed procedures for DCE-MRI data processing have been previously described (Davoodi-Bojd et al., 2019). The processing begins with the correction of motion that occurs during the 5-h MRI scan, followed by the co-registration of all animal data to a standard reference template so that comparisons between groups can be carried out in a common spatial space. To detect differences in glymphatic tracer transport in the AD brain with and without CEC-Exo treatment, tracer-induced time signal curves (TSCs) within representative brain regions that represent the dynamic tracer uptake and clearance in the tissue regions were measured.

We employed our in-house MATLAB-based software to process dMRI data, including motion correction, tensor modeling, and the calculation of scalar maps. Using this software, dMRI data were processed based on their *b*-values. By fitting the DWI signals at high *b*-values (*b* = 900, 1,800 s/mm^2^), diffusion parameters (derived from the Gaussian (monoexponential) and non-Gaussian (Kurtosis) diffusion models) sensitive to slow diffusion in the brain parenchyma, such as axonal water fraction (AWF), fractional anisotropy (FA), Kurtosis, mean diffusivity (MD), axial diffusivity (AD), and radial diffusivity (RD), were estimated, while DWI data acquired at low *b*-values (*b* = 50, 80, 120, 150, 180 s/mm^2^) were employed to estimate mean diffusivity (MD_low-b_, Gaussian diffusion model) that reflects fast pseudo-diffusion in the CSF system (Rane Levendovszky et al., 2024; Voorter et al., 2025). To detect alterations in microstructural integrity in the brain parenchyma and CSF motion in the SAS resulting from CEC-Exo therapy, these diffusion parameters were evaluated in representative brain regions and CSF-filled space, respectively.

### 2.3 Cognitive performance

To assess spatial learning acquisition and memory retention, a modified Morris water maze (MWM) test (Darwish et al., 2012; Li et al., 2019) was performed. The testing system consisted of a circular tank (180 cm in diameter and 45 cm high) filled with water maintained at 30 °C and a hidden platform (15 cm in diameter and 35 cm high, transparent and invisible to animals) set inside the tank (1.5 cm below the water surface). The pool was in a large test room decorated with visual spatial clues (e.g., lamps, pictures on the walls, and a camera on the ceiling) that remained constant during the test, enabling the rats to orient themselves spatially. For descriptive data collection, an automated tracking system (HVS Image, San Diego, CA) was employed and the pool was subdivided into four equal quadrants formed by imaging lines. In the last week of the 13th month, the rats were tested for five consecutive days, four trials per day with an inter-trial interval of 30 min. Each trial was initiated by placing the animal (facing the wall of the pool) randomly at one of four fixed start locations (designated North, South, East, and West) and allowing 90s for the animal to find the submerged platform that was placed in a randomly changing position within the northeast (NE) quadrant throughout the five-day testing period. After locating the platform, the animal was allowed to remain on the platform for 15 s before being returned to its cage. If the animal failed to find the platform within 90s, the experiment was terminated and a maximum score of 90s was assigned. In such cases, the animal was guided to the platform and allowed for 15 s of exploration before being returned to its cage. The percentage of time spent in the NE (correct) quadrant was calculated relative to the total swimming time before reaching the platform. Data were averaged over four trials each day to yield a single value for each rat per day.

### 2.4 Quantification and statistical analysis

Referring to the anatomical atlas, all regions of interest (ROIs) were manually delineated over specific sagittal or coronal image slices in a blinded fashion using MRIcro and ImageJ.

**Figure 1:**
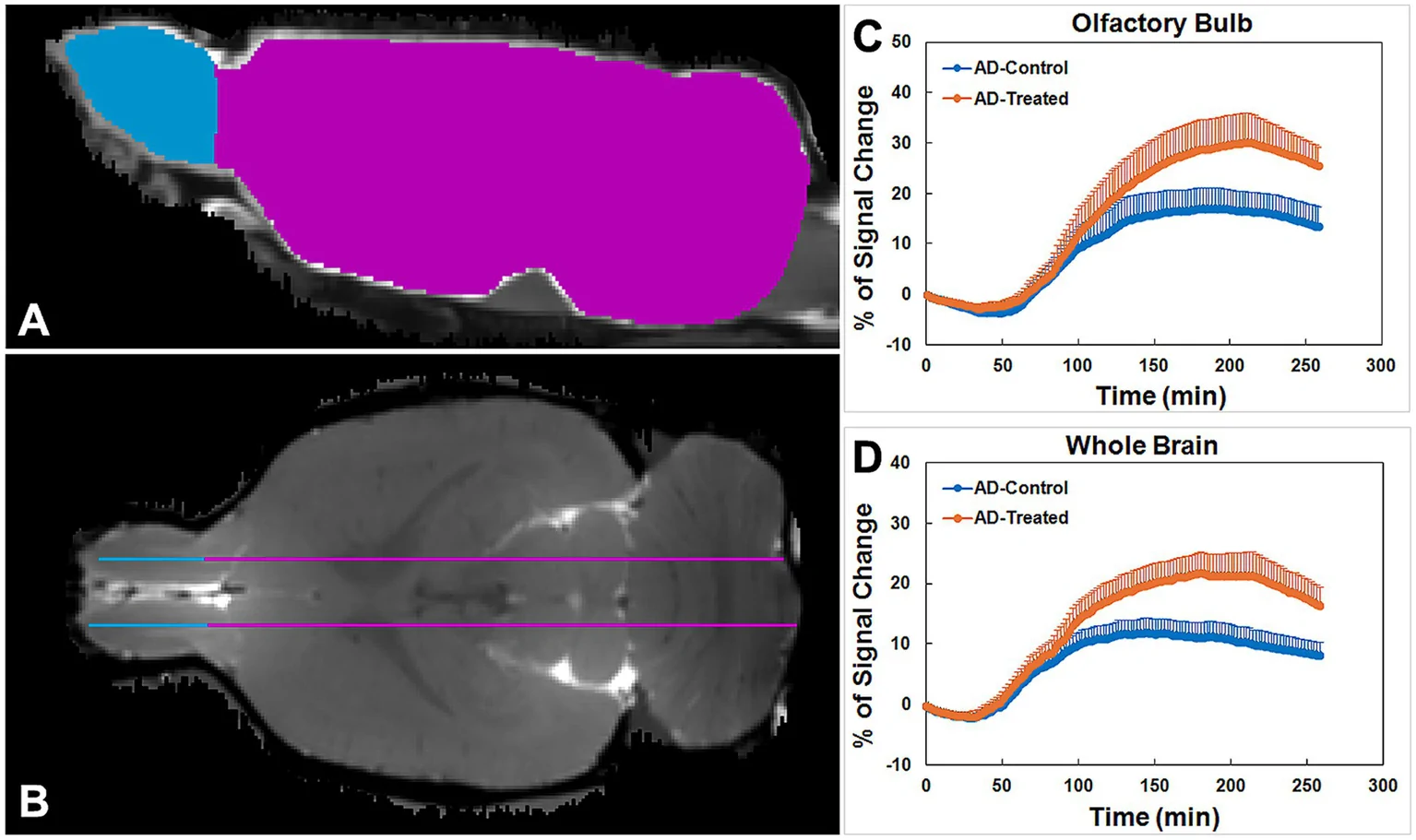
ROIs, TSCs, and the effect of CEC-Exo therapy on glymphatic transport. ROIs encompassing the olfactory bulb (blue area in **A**) and the whole brain (blue area plus pink area in **A**) were generated on sagittal sections (**A**, lateral 0.40 mm) in two hemispheres of the brain symmetrically **(A, B)**. The TSCs measured from these ROIs **(C, D)** showed that, during most of the observation period, higher percentages of signal change in both the olfactory bulb **(C)** and whole brain **(D)** were exhibited in the AD-Treated group than in the AD-Control group, with significant differences in peak values of TSCs detected between the groups (olfactory bulb: 33.73 ± 5.20 vs. 19.47 ± 3.87, *p* = 0.04; whole brain: 24.09 ± 3.50 vs. 13.73 ± 2.17, *p* = 0.02).

To measure the TSCs that characterize the kinetic features of glymphatic tracer transport (Figure 1), ROIs encompassing the olfactory bulb (blue area in Figure 1A) and whole brain (blue area plus pink area in Figure 1A) were generated on sagittal sections (Figure 1A, Lateral 0.40 mm) symmetrically in both hemispheres of the brain (Figures 1A,B).

**Figure 2:**
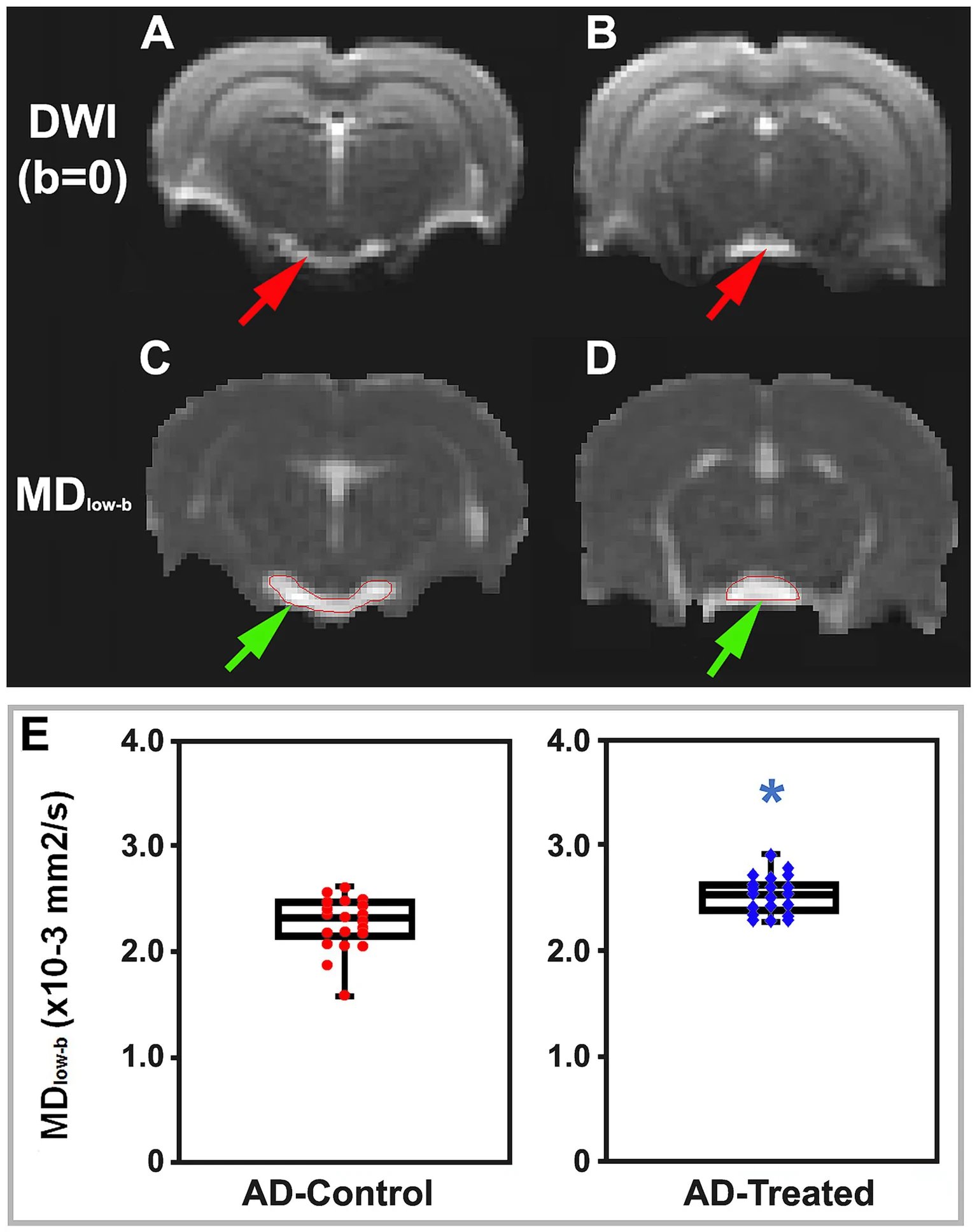
ROIs for evaluation of CSF motion in the representative anatomical CSF-filled space and the effect of CEC-Exo therapy on CSF MD_low-b_. As indicated by red arrows in **(A,B)**, the interpeduncular cistern located at the ventral side of the brain was represented by bright pixels on two contiguous DWI (*b* = 0) slices (approximately bregma −5 and −6 mm, respectively). ROIs encompassing these bright regions were delineated for each animal and copied onto the corresponding slices of MD_low-b_ map (**C**,**D**, red closed curves indicated by green arrows) for quantitative evaluation. Group results **(E)** demonstrated a significantly increased MD_low-b_ in the AD-Treated rats compared with the AD-Control rats (2.55 ± 0.04 vs. 2.26 ± 0.06, *p* < 0.001).

To evaluate CSF motion, ROIs enclosing the representative CSF-filled space were created. As shown in Figure 2, the interpeduncular cistern around the ventral surface of the brain was characterized by bright pixels (red arrows in Figures 2A,B) on two contiguous coronal DWI (*b* = 0) slices (approximate bregma −5 and −6 mm, respectively). ROIs encompassing these bright fluid-filled regions were delineated for each animal and copied onto the corresponding slices of the MD_low-b_ map (red closed curves indicated by green arrows in Figures 2C,D) for quantification.

**Figure 3:**
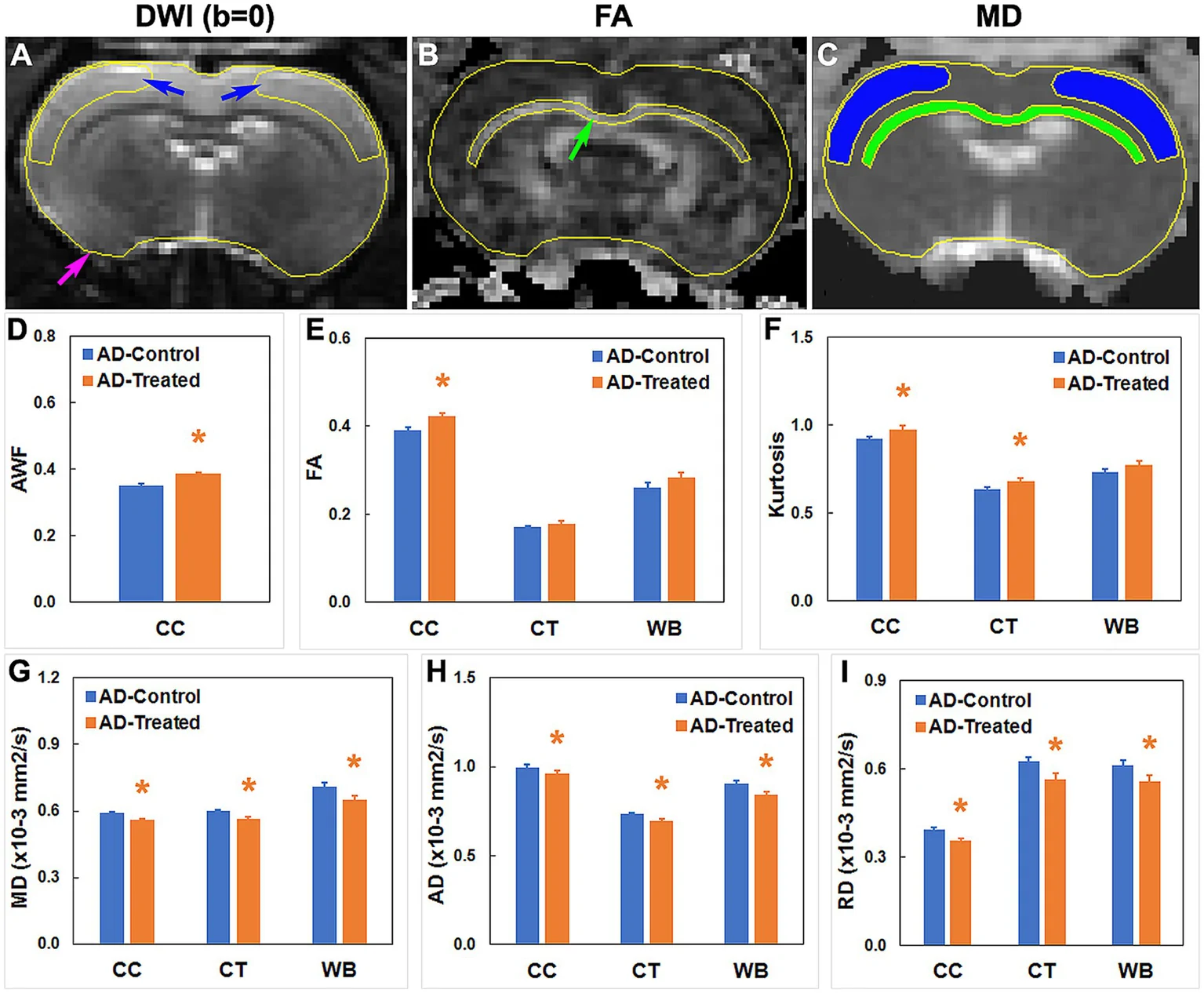
ROIs for diffusion evaluation of brain parenchyma and the effect of CEC-Exo therapy on cerebral microstructural integrity. On a fixed coronal slice (approximately bregma −1 mm), ROIs encompassing the whole brain (WB, pink arrow in **A**) and cortex (CT, blue arrows in **A**) were drawn on DWI (*b* = 0) **(A)**, while the ROI encompassing the corpus callosum (CC, green arrow in **B**) was delineated on the FA map **(B)**. These ROIs were copied onto the corresponding slice of parametric maps, such as MD **(C)**, for quantification of diffusion features in each ROI. Compared with the AD-Control group, significantly higher AWF **(D)**, FA **(E)**, and Kurtosis **(F)** values in the CC, and significantly lower MD **(G)**, AD **(H)**, and RD **(I)** values in the CC, CT, and WB were found in the AD-Treated group (Detailed data were listed in Table 1).

To assess the cerebral microstructural integrity, ROIs focusing on specific structures (e.g., white matter and gray matter) were made. On a fixed coronal slice (approximate bregma −1 mm), ROIs encompassing cortex (CT) areas on both hemispheres of the brain were drawn on DWI (*b* = 0) (blue arrows in Figure 3A), while an ROI covering the corpus callosum (CC) was created on FA map (green arrow in Figure 3B). Due to the small size and narrow shape of the CC region, special care was taken during its delineation to minimize the contamination from surrounding tissues. ROI encompassing the whole brain (WB), including various structures and ventricles, was also created for completeness. As shown in Figure 3C, superimposed is the complete set of ROIs utilized for quantitative evaluation of diffusion features in the brain parenchyma.

Based on the effect size observed in our pilot MRI data (Cohen’s *d* = 1.32), an *a priori* power analysis for a two-sided independent samples *t*-test indicated that a sample size of n = 10 rats per group (total *N* = 20) is required to achieve 80% statistical power with an alpha level of 0.05.

To detect the effects of CEC-Exo therapy on glymphatic transport revealed by regional TSCs, CSF motion evaluated by MD_low-b_, and microstructural integrity characterized by dMRI_high-b_-derived parameters, a two-sample *t*-test was performed between groups. Mixed-design ANOVA was employed to evaluate group differences in cognitive performance, assessed longitudinally using the MWM, with treatment group as the between-subjects factor and time as the within-subjects factor. The analysis began by testing the group × time interaction. If a significant interaction was detected (alpha = 0.05), this was followed by tests of simple main effects to determine group differences at each time point and the effect of time within each treatment group. In the absence of significant interaction, the main effects of treatment group and time were evaluated. If neither the interaction nor the main effects were detected at the 0.05 level, any subgroup analyses were considered exploratory. Statistical significance was set at *p* < 0.05 and data were expressed as the mean ± standard error.

## 3 Results

### 3.1 Effect of CEC-Exo therapy on glymphatic transport

The TSCs obtained from specific brain areas represent the retention of infused CSF tracer as a function of time in these tissue regions, thereby reflecting the kinetic features of glymphatic transport. Based on the TSC, glymphatic tracer transport is mainly characterized by tracer-induced signal change, signal accumulation, and signal attenuation over time, which correspond to the amount of tracer uptake, tracer infusion, and tracer clearance, respectively (Davoodi-Bojd et al., 2019; Li et al., 2022a). The comparison of regional TSCs between groups is shown in Figure 1. During most of the observation period, higher percentages of signal change in the olfactory bulb (Figure 1C) and whole brain (Figure 1D) were exhibited in the AD-Treated rats than in the AD-Control rats with significant differences detected in the peak TSC values in both the olfactory bulb (33.73 ± 5.20 vs. 19.47 ± 3.87, *p* = 0.04) and whole brain (24.09 ± 3.50 vs. 13.73 ± 2.17, *p* = 0.02). Increased rates of signal accumulation and signal attenuation were also observed in these examined regions in the AD-Treated group compared to the AD-Control group, although these differences did not reach statistical significance. These data suggest that CEC-Exo treatment induces an increased amount of CSF passing through the glymphatic system in the AD brain, demonstrating enhanced glymphatic transport function.

### 3.2 Effect of CEC-Exo therapy on CSF motion

Using MD_low-b_, CSF motion within the SAS of the brain was evaluated in the interpeduncular cistern (Figure 2). Compared with the AD-Control rats, a significantly increased MD_low-b_ was detected in the AD-Treated rats (Figure 2E, 2.55 ± 0.04 vs. 2.26 ± 0.06, *p* < 0.001), indicating improved CSF motion because of the CEC-Exo therapy. Furthermore, our diffusion and glymphatic measurements demonstrated that, concurrent with the improvement of CSF motion (Figure 2), there was an enhancement of glymphatic transport (Figure 1), representing consistent therapeutic responses to CEC-Exo intervention. These data suggest that MD_low-b_ is sensitive to treatment-induced increase in CSF motion in the SAS, which is associated with augmentation of glymphatic function in the brain parenchyma.

### 3.3 Effect of CEC-Exo therapy on cerebral structural integrity

Diffusion features in white matter (CC), gray matter (CT) and whole brain (WB) were evaluated in the AD brain with and without CEC-Exo therapy. Our data (Table 1, Figure 3) showed that significantly higher AWF (Figure 3D), FA (Figure 3E), and Kurtosis (Figure 3F) in the CC were detected in the AD-Treated group than in the AD-Control group, suggesting amelioration of white matter damage in the AD brain with CEC-Exo treatment. Our results also showed that significantly lower MD (Figure 3G), AD (Figure 3H), and RD (Figure 3I) in CC, CT, and WB were found in the AD-Treated group than in the AD-Control group, indicating ameliorated microstructural disruption not only in white matter but also in gray matter because of the CEC-Exo treatment.

**Table 1:** Group comparisons of diffusion parameters derived from dMRI_high-b_ (mean ± SE).

Diffusion parameters	Saline-treated			CEC-Exo-treated		
	CC	CT	WB	CC (*p* value)	CT (*p* value)	WB (*p* value)
AWF	0.350 ± 0.012			0.388 ± 0.012 (0.02)		
FA	0.382 ± 0.008	0.171 ± 0.006	0.249 ± 0.012	0.424 ± 0.010 (0.002)	0.178 ± 0.005 (0.2)	0.268 ± 0.012 (0.1)
Kurtosis	0.926 ± 0.020	0.640 ± 0.012	0.726 ± 0.021	0.989 ± 0.019 (0.02)	0.684 ± 0.015 (0.02)	0.776 ± 0.023 (0.06)
MD	0.592 ± 0.009	0.604 ± 0.006	0.714 ± 0.016	0.559 ± 0.008 (0.01)	0.563 ± 0.011 (0.002)	0.662 ± 0.017 (0.02)
AD	0.998 ± 0.014	0.744 ± 0.015	0.907 ± 0.015	0.952 ± 0.013 (0.01)	0.686 ± 0.011 (0.003)	0.840 ± 0.014 (0.002)
RD	0.392 ± 0.010	0.634 ± 0.017	0.617 ± 0.018	0.356 ± 0.007 (0.006)	0.559 ± 0.022 (0.007)	0.566 ± 0.017 (0.02)

*p* value: AD rats with CEC-Exo treatment vs. AD rats with saline treatment in the same brain region. CC: corpus callosum; CT: cortex; WB: whole brain.

### 3.4 Effect of CEC-Exo therapy on cognitive function

**Figure 4:**
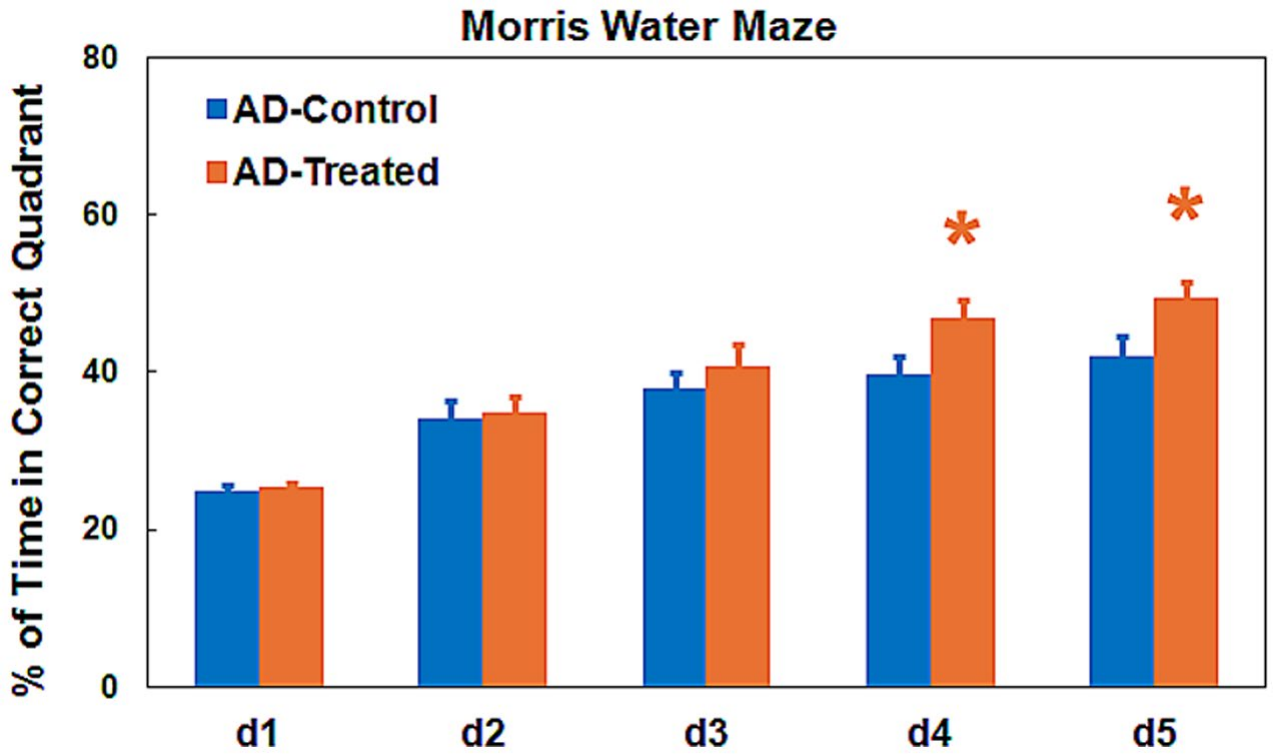
The effect of CEC-Exo therapy on cognitive task performance. As assessed by the MWM test, AD rats with CEC-Exo treatment spent a significantly greater percentage of time in the correct quadrant compared with AD rats with saline treatment (d4: 46.82 ± 2.33 vs. 39.66 ± 2.27, *p* = 0.04; d5: 49.47 ± 2.11 vs. 42.07 ± 2.60, *p* = 0.04).

As detected by the MWM test (Figure 4), CEC-Exo therapy led to a significant improvement in cognitive task performance (Figure 4, d4: 46.82 ± 2.33 vs. 39.66 ± 2.27, *p* = 0.04; d5: 49.47 ± 2.11 vs. 42.07 ± 2.60, *p* = 0.04). The ameliorated cerebrovascular dysfunction (Li et al., 2021; Ding et al., 2022; Zhang et al., 2022), enhanced glymphatic transport (Figure 1), and preserved microstructural integrity (Figure 3) after CEC-Exo treatment may collectively contribute to the improved cognitive function.

## 4 Discussion

Using DCE-MRI and dMRI, we, for the first time, investigated the effect of CEC-Exo therapy on AD as indicated by glymphatic transport, CSF motion, and microstructural integrity. Alterations in glymphatic function were assessed with tracer-induced TSCs, while changes in subarachnoid CSF motion and cerebral microstructural state were evaluated with dMRI_low-b_- and dMRI_high-b_-derived parameters, respectively. Our study demonstrated that CEC-Exo intervention after AD led to enhanced glymphatic transport, increased CSF motion, reduced microstructural disruption, and improved cognitive function. With consistent beneficial outcomes detected in both glymphatic transport and CSF motion, the results evidence their relevance and support the potential value of dMRI_low-b_ as a clinically feasible imaging tool for assessing the alterations in CSF dynamics.

Neurovascular dysfunction is strongly implicated in the pathogenesis of AD (Solis et al., 2020; Soto-Rojas et al., 2021; Szu and Obenaus, 2021; Fisher et al., 2022; Alkhalifa et al., 2023). Changes in the cerebral vasculature initiate a vicious cycle between brain Aβ accumulation and neurovascular unit impairment, leading to derangement of perivascular fluid movement and compromise of glymphatic transport (Kyrtsos and Baras, 2015; Reeves et al., 2020; Ang et al., 2024). As both a causative and consequent contributor to AD pathogenesis, increasing evidence underscores the critical importance of the cerebrovascular condition in preventing and treating AD (Yamazaki and Kanekiyo, 2017; Fisher et al., 2022; Tang et al., 2025). Our previous studies demonstrate that treatment of stroke and diabetes with CEC-Exo ameliorates cerebrovascular damage and dysfunction (Li et al., 2021; Ding et al., 2022; Zhang et al., 2022), which may contribute to the improved glymphatic transport observed in the context of AD (Figure 1). As characterized by the TSCs, the significantly elevated percentages of signal change were accompanied by increased rates of signal accumulation and signal attenuation after treatment, indicating augmented periarterial tracer infusion and perivenous tracer clearance, respectively. As a key factor in AD progression, impaired cerebral waste clearance creates a toxic environment that drives cerebral microstructural degeneration in the AD brain, which in turn results in cognitive decline (Peng et al., 2016; Lin et al., 2022; Hong et al., 2024). In support of the critical role of cerebral waste clearance in AD, our study showed that the enhanced glymphatic clearance function after CEC-Exo intervention (Figure 1) was associated with the reduced cerebral microstructural disruption (Figure 3) and improved cognitive performance (Figure 4). Despite the lack of effective strategies to halt AD progression (Sousa et al., 2023), our data indicate that CEC-Exo therapy offers a promising new direction by targeting fundamental aspects of the disease, such as neurovascular health (Li et al., 2021; Ding et al., 2022; Zhang et al., 2022) and cerebral waste clearance (Figure 1).

As a major player in clearing waste products from the CNS, CSF movement in the SAS is tightly linked to solute influx into and efflux from the brain parenchyma (Hornkjøl et al., 2022). While the major arteries and cisterns present the preferential pathway along which SAS CSF enters and recirculates back to the brain (Bedussi et al., 2017), prior work demonstrates that, compared with other SAS regions, stronger solute transport occurs at the ventral part of the brain, where large arteries (e.g., the circle of Willis) and cisterns (e.g., basal cisterns) reside (Iliff et al., 2013; Bedussi et al., 2017; Li et al., 2022a). Furthermore, a previous study reveals a functional connection between the ventricular system and the SAS via the interpeduncular cistern (Bedussi et al., 2017). This basal cistern, containing major arteries and the basal vein of Rosenthal implicated in fluid movement, therefore represents a logical location for assessing CSF motion in the SAS that relates to CSF dynamics in the brain. To quantify the variation in CSF motion, the region of the interpeduncular cistern was selected in the current study, which can be identified (as bright fluid-filled areas) on two contiguous DWI (*b* = 0) slices (Figure 2) in our measurement setting. Concurrent with an enhanced glymphatic transport found in the brain parenchyma (Figure 1), an increased CSF motion was detected in the SAS (Figure 2) because of CEC-Exo treatment. These consistent therapeutic effects demonstrate a continuous CSF compartment, with CSF motion in the SAS being relevant to perivascular flow in the brain parenchyma. These results also demonstrated the sensitivity of dMRI_low-b_ to variations in subarachnoid CSF motion that were associated with alterations in cerebral glymphatic transport. In line with and expanding upon previous finding (Tazoe et al., 2023), our study showed that the basal cistern (e.g., interpeduncular cistern) of the rodent brain represents an important location to evaluate alterations in CSF dynamics caused by either brain injury (Tazoe et al., 2023) or therapeutic intervention (Figure 2). While DCE-MRI provided regional kinetic features of glymphatic transport in the brain parenchyma (Iliff et al., 2013; Jiang et al., 2017; Li et al., 2022a), it was performed under non-physiological condition (Matsumae et al., 2019) due to the requirement of tracer delivery (Iliff et al., 2013; Jiang et al., 2017; Ding et al., 2018; Davoodi-Bojd et al., 2019). In addition, repeated DCE-MRI measurements at multiple time points are also needed to obtain the temporal profiles of both tracer infusion and tracer clearance, which take at least over 24-h’ time span for human brain after intrathecal tracer injection (Ringstad et al., 2017; Ringstad et al., 2018; Yamamoto et al., 2024) and cannot be translated to the clinic. Without CSF tracer administration, and therefore without the induced disturbance and invasiveness, our data demonstrated that dMRI_low-b_ offers a measure of CSF motion in the representative anatomical CSF-filled space that is linked to perivascular glymphatic transport. As a non-invasive quantitative method with operational advantages over DCE-MRI, the results of this study thus support the value of dMRI_low-b_ as a clinically feasible imaging tool for assessing changes in CSF dynamics that reflect alterations in glymphatic transport.

By revealing abnormalities in water diffusion patterns, dMRI_high-b_ provides valuable insights into brain tissue microstructure. As markers of cerebral microstructure, FA, AWF, and Kurtosis characterize the directionality of water diffusion, the proportion of water confined within axons, and the state of restricted diffusion in tissues, respectively (Li et al., 2019; Dong et al., 2020), with FA and AWF being most applicable to well-defined white matter tracts (Benitez et al., 2014; Li et al., 2019). Higher values of these parameters, therefore, indicate more organized and directed white matter fiber bundles, larger axonal volume and density, and greater heterogeneity and complexity of tissue microstructure, respectively. Reduced brain tissue integrity, such as axonal injury and myelin damage, which alter the restricted diffusion state, can be featured by increased MD, AD, and RD that reflect the diffusion rate of water molecules in all directions, along and perpendicular to the main direction of the water movement, respectively (Dong et al., 2020; Collij et al., 2021). Microstructural disruption involves both white matter (Dong et al., 2020; Collij et al., 2021) and gray matter (Debatisse et al., 2025; Zhao et al., 2025) in the AD brain. Our evaluation and analysis, therefore, mainly focused on the corpus callosum (CC) and cortex (CT), the representative regions of white matter and gray matter in the rat brain, respectively. With CEC-Exo therapy, amelioration of white matter damage was found in the AD brain, as evidenced by significantly higher AWF, FA, and Kurtosis values in the CC in the AD-Treated group than in the AD-Control group (Figures 3D–F). Furthermore, significantly lower MD, AD, and RD values in the CC and CT were detected in the AD brain after treatment (Figures 3G–I), indicating ameliorated microstructural disruption not only in white matter but also in gray matter. The increased microstructural integrity likely benefited from the therapy-enhanced cerebrovascular function (Li et al., 2021; Ding et al., 2022; Zhang et al., 2022), which contributed to augmented perivascular waste clearance (Figure 1), thereby potentially reducing Aβ plaques and the corresponding microstructural degeneration (Snow et al., 2017; Dong et al., 2020; Collij et al., 2021; Zhao et al., 2025). Previous studies have shown that glymphatic dysfunction in the AD brain is associated with both vascular deficits and Aβ burden (Hong et al., 2024), whereas slowed Aβ aggregation, reduced Aβ pathology, and attenuated neuroinflammation after exosome-based therapies accompany sustained microstructural integrity (Webb et al., 2018; Xu et al., 2020; Yin et al., 2023; Zhou et al., 2024). Given the improved glymphatic transport (Figure 1), which could exert a positive influence, these exosome-induced therapeutic mechanisms may underlie the preserved microstructural integrity (Figure 3) observed in the present study. Moreover, cerebrovascular dysfunction significantly contributes to cognitive impairment (Snyder et al., 2015; Kapasi and Schneider, 2016; Graff-Radford, 2019; Tang et al., 2025). Aβ and tau pathologies, typical AD biomarkers, represent well-established correlates of cognitive disabilities (King-Robson et al., 2021; Ge et al., 2022; Tang et al., 2025). The reduced cognitive decline after CEC-Exo treatment (Figure 4) may therefore be attributed to ameliorated vascular deficits (Li et al., 2021; Ding et al., 2022; Zhang et al., 2022) as well as the rescued brain function, such as the preserved glymphatic transport and CSF motion (Figures 1, 2), that facilitated cerebral waste clearance and reduced brain microstructural damage (Figure 3). Causality linking enhanced glymphatic function to cognitive improvement needs further experimental validation.

Brain injury alters the diffusion characteristics in both the brain parenchyma and CSF system (Tazoe et al., 2023). The altered diffusion parameters likely also reflect the amelioration of cerebral microstructural disruption (Figure 3) and CSF motion abnormalities (Figure 2) after CEC-Exo treatment, illustrating the power of dMRI as an imaging tool for detecting structural and functional changes in response to brain insult and therapeutic intervention.

The strengths of this study include the *in vivo* imaging of therapeutic effects concomitantly represented by glymphatic transport and CSF motion in the AD brain and the use of a rat model with larger brain and SAS than the mouse that facilitate ROI identification and regional evaluation. There are limitations in this study. First, we performed a cross-sectional investigation due to the invasive surgery required for glymphatic MRI. However, our data demonstrated that a longitudinal study could be conducted using non-invasive dMRI_low-b_ to reveal alterations in CSF dynamics that are linked to variations in glymphatic transport. Second, we included both sexes with equal numbers of males and females (5 vs. 5) in each experimental group. Although no intragroup sex differences were observed in all measurements, conclusions regarding sex effects cannot be drawn based on this small sample size of certain sex in each group. Given the previous findings that show evident sex differences across various aspects in animal models (Jeong et al., 2023; Ocañas et al., 2023; Robison et al., 2023) and human AD (Bergamino et al., 2022; Aggarwal and Mielke, 2023; Nazeri et al., 2025), further investigation with specific experimental design for sex-dependent comparison is needed. Finally, we treated TgF344-AD rats at the age of 12-months when a repertoire of AD pathological features, such as neurovascular dysfunction (Joo et al., 2017), A*β* deposition and cognitive impairment (Cohen et al., 2013), developed. An in-depth study on treatment strategy (e.g., the timing of initial treatment, consecutive treatment period and dosage) with longitudinal observation would reveal more complete information about the effects of CEC-Exo therapy on AD. Further studies on investigating and optimizing CEC-Exo treatment protocols for AD are warranted. Consistent with current understanding of AD pathophysiology, we linked the amelioration of cerebrovascular dysfunction to improved glymphatic transport, which was associated with reduced Aβ pathology and cognitive decline. However, future studies will be necessary to incorporate direct vascular readouts and Aβ pathology evaluations, which were lacking in the current investigation.

Exo-based strategies including CEC-Exo have emerged as promising therapeutic agents for various neurological diseases due to their unique properties, including low immunogenicity, intrinsic biocompatibility, and capacity to bypass the BBB (Alvarez-Erviti et al., 2011; Kalluri and LeBleu, 2020; Zhang et al., 2022). CEC-Exo are particularly attractive for AD therapy, as they can efficiently deliver bioactive cargo to the CNS without the limitations typically associated with conventional AD drugs, such as limited BBB permeation and unwanted gastrointestinal side effects (Singh et al., 2024). Our prior work has established that intravenously administered CEC-Exo cross the BBB and are internalized by cerebral endothelial cells and neurons (Zhang et al., 2022), confirming delivery to the brain parenchyma. However, it remains unclear whether CEC-Exo directly interact with cells along the cerebral vasculature and glymphatic pathways, particularly astrocytes expressing aquaporin-4 (AQP4) channels, which act as critical gatekeepers of glymphatic transport. Although CEC-Exo are enriched with bioactive cargo and alleviate neurovascular damage by modulating regulatory miRNAs and proteins involved in BBB maintenance and vascular function, the specific cargo molecules and their interactions with target cells in the AD brain have not been addressed. This limitation significantly hinders our ability to define the precise molecular mechanisms underlying the observed therapeutic benefits. Furthermore, substantial translation barriers remain regarding delivery, dosage, and immune compatibility. To guide clinical translation, optimal doses, treatment frequencies, and administration routes must be carefully evaluated to maximize brain bioavailability while minimizing peripheral off-target effects. This requires rigorous safety assessments and biodistribution studies in preclinical models. Additionally, caution must be exercised as treatment protocols derived from animal models may not translate directly to humans. Therefore, appropriate therapeutic regimens for potential human studies remain to be established. Despite these challenges and caveats, the robust beneficial effects of CEC-Exo on glymphatic transport, CSF motion, microstructural integrity, and cognitive function support the continued development of CEC-Exo-based strategies for AD. Future studies are needed to elucidate the molecular cargo and target cell interactions including analysis of tight junction proteins and AQP4 expression, and to optimize therapeutic regimens prior to clinical translation.

In summary, our study demonstrated a novel exosome-based CEC-Exo therapy that preserved glymphatic transport, CSF motion, and microstructural integrity in the AD brain. With CEC-Exo treatment, a brain-wide amelioration of glymphatic dysfunction was obtained. Meanwhile, reduced deterioration of cerebral microstructure was observed in both white matter and gray matter, where destructive structural changes occur in the AD brain. The therapy, therefore, had a global impact on both the function and structure of the AD brain. The observed outcomes may be, at least in part, attributed to CEC-Exo-induced improvement of cerebrovascular function, which beneficially affects perivascular glymphatic transport and, therefore, toxic protein buildup and the corresponding microstructural state. Our DCE-MRI and dMRI_low-b_ detected consistent therapeutic effects of CEC-Exo intervention on glymphatic transport in the brain parenchyma and on CSF motion in the SAS, evidencing their relevance. While DCE-MRI holds promise as a reliable means for detecting glymphatic tracer transport in the brain parenchyma, our data demonstrated the value of dMRI_low-b_ as a non-invasive, non-disruptive method for assessing alterations in CSF dynamics that mediate perivascular glymphatic clearance.

## Data Availability

The original contributions presented in the study are included in the article/supplementary material, further inquiries can be directed to the corresponding author.
